# Optically Programmable Living Microrouter in Vivo

**DOI:** 10.1002/advs.202304103

**Published:** 2023-09-25

**Authors:** Xiaoshuai Liu, Huaying Wu, Shuai Wu, Haifeng Qin, Tiange Zhang, Yufeng Lin, Xianchuang Zheng, Baojun Li

**Affiliations:** ^1^ Guangdong Provincial Key Laboratory of Nanophotonic Manipulation, Institute of Nanophotonics Jinan University Guangzhou 511443 China

**Keywords:** drug delivery, nanotherapeutics, optical tweezers, programmable medical micromachines, red blood cells

## Abstract

With high reconfigurability and swarming intelligence, programmable medical micromachines (PMMs) represent a revolution in microrobots for executing complex coordinated tasks, especially for dynamic routing of various targets along their respective routes. However, it is difficult to achieve a biocompatible implantation into the body due to their exogenous building blocks. Herein, a living microrouter based on an organic integration of endogenous red blood cells (RBCs), programmable scanning optical tweezers and flexible optofluidic strategy is reported. By harvesting energy from a designed optical force landscape, five RBCs are optically rotated in a controlled velocity and direction, under which, a specific actuation flow is achieved to exert the well‐defined hydrodynamic forces on various biological targets, thus enabling a selective routing by integrating three successive functions, i.e., dynamic input, inner processing, and controlled output. Benefited from the optofluidic manipulation, various blood cells, such as the platelets and white blood cells, are transported toward the damaged vessel and cell debris for the dynamic hemostasis and targeted clearance, respectively. Moreover, the microrouter enables a precise transport of nanodrugs for active and targeted delivery in a large quantity. The proposed RBC microrouter might provide a biocompatible medical platform for cell separation, drug delivery, and immunotherapy.

## Graphical abstract

By integrating the programmable scanning optical tweezers and flexible optofluidic strategy, a living microrouter is designed and constructed from endogenous red blood cells for the desired routing of various biological targets while toward their respective destinations in vivo, which may provide an intelligent, multifunctional, and programmable medical platform for cell separation, drug delivery, and immunotherapy.

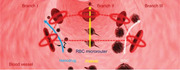

## 1 Introduction

Dynamic routing of various blood components in vivo, i.e., directing them along their respective routes, offers great potentials for a series of biomedical applications ranging from cell separation, drug delivery, to medical diagnosis, immunotherapy and clinical surgery.^[^ ^1^, ^2^, ^3^, ^4^ ^]^ For this purpose, programmable medical micromachines (PMMs), inspired by the cooperation behavior in the biological world such as bird flocks and insect swarms, are emerging as a promising strategy because of their micron size, active propulsion, and wireless remote control.^[^ ^5^, ^6^, ^7^ ^]^ Moreover, benefited from their high reconfigurability and swarming intelligence, the interaction among the building blocks of PMMs can be precisely tailored in response to various environmental stimuli, thus providing excellent programming and tuning capabilities to execute desired coordinated tasks.^[^ ^8^, ^9^, ^10^, ^11^ ^]^

Over the last few decades, through the integration of micro/nano‐fabrication techniques and external field induced interactions, a variety of PMMs have been developed for dynamic routing of objects.^[^ ^12^, ^13^, ^14^ ^]^ For example, a dynamic routing was achieved for various artificial bacterial flagella (ABFs) toward different branches, by setting the rotating frequency of the external magnetic field below the step‐out frequencies of selected ABFs but higher than the step‐out frequencies of unselected ones.^[^ ^15^ ^]^ Although these artificial devices have achieved considerable success under in vitro conditions, they usually face challenges for further in vivo applications due to the issue of biocompatibility or biodegradability. Recently, many biological cells, such as Escherichia coli,^[^ ^16^ ^]^ microalgae,^[^ ^17^, ^18^ ^]^ and yeast cells,^[^ ^19^ ^]^ have been utilized as natural building blocks to assemble the PMMs, thus bridging the gap between synthetic machine and biological world.^[^ ^20^, ^21^ ^]^ However, due to their foreign nature, invasive implantation will be required to introduce them into the body, which may trigger unwanted immune response. The assembly of biocompatible PMMs based on endogenous cells remains to be achieved for precise routing in vivo.

As the most abundant blood cells in the circulatory system, red blood cells (RBCs) can be excellent candidates for constructing endogenous PMMs because of their perfect biocompatibility, long circulation, non‐immunogenicity, and inert intracellular environment.^[^ ^22^, ^23^, ^24^ ^]^ In addition, their biological motions can be handled by external field, e.g., the optical force can manipulate the movement of RBCs in a noncontact and noninvasive manner with single‐cell precision.^[^ ^25^, ^26^ ^]^ For example, by exploiting the optical force exerted on the RBCs, especially for the forward‐scattering and gradient forces, Chen et al. first demonstrated the nonlinear self‐trapping of light over centimeter propagation distances through scattering RBC suspensions, which might provide a powerful photonic tool for noninvasive biomedical imaging and medical diagnosis.^[^ ^27^, ^28^ ^]^ In this work, a living microrouter has been constructed by integrating endogenous RBCs, programmable scanning optical tweezers (SOTs) and flexible optofluidic strategy in an organic manner. By scanning the beam focus in a time‐shared manner, a desired optical force landscape can be generated to arrange five optically trapped RBCs into a pentagon, followed by a dynamic rotation of the RBCs with controlled velocities and directions. Notably, each rotating RBC can be regarded as one endogenous microrotor.^[^ ^29^ ^]^ Under the actuation of five microrotors, a desired microflow field will be induced in the surrounding area. By sculpting the microflow field in a programmable manner, a selective routing capacity is achieved through the combination of three successive functions, i.e., dynamic input, inner processing, and controlled output. The obtained living microrouter is applicable for various biological targets, such as the platelets and white blood cells (WBCs), which were directed toward damaged vessel and cell debris for controlled hemostasis and targeted clearance, respectively. Furthermore, the desired routing for the nanodrugs was demonstrated in a large quantity, thus providing a high‐precision and programmable strategy for the active and targeted drug delivery in vivo.

## 2 Results

### 2.1 The design Principle and Numerical Simulation of the RBC Microrouter

**Figure 1:**
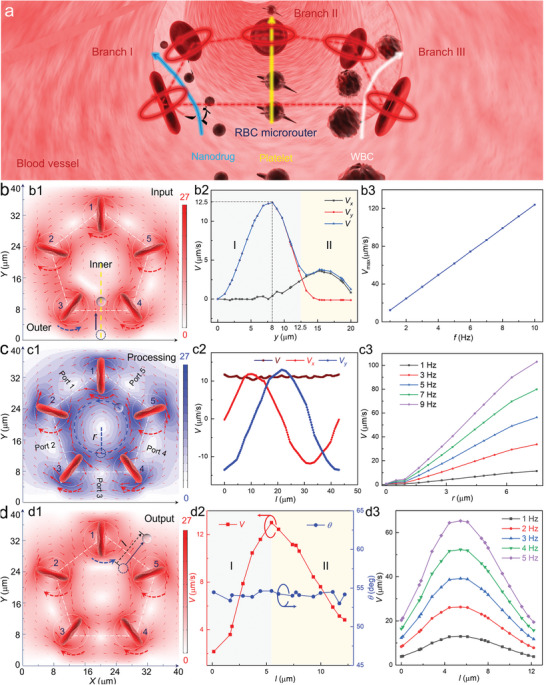
a) Schematic illustration for exploiting the RBC microrouter to selectively route various targets into designed branches. b) Simulated microflow field distribution for target input. b1) Importing the target from port 3 with the velocity vectors indicated by the red solid arrows. b2) The calculated velocity as a function of *y* for the target input along the yellow dashed line in b1. b3) The calculated input velocity as a function of *f*. c) Simulated microflow field distribution for inner processing. c1) The target was rotated inside RBC microrouter. The velocity contour surface and microflow direction are indicated by the blue surface and red solid arrows, respectively. c2) The calculated velocity for the rotation of the target along the blue dashed circle. c3) The calculated velocity as a function of rotation radius *r* (blue dashed line) under various rotation frequency. d) Simulated microflow field distribution for target output. d1) Exporting the target from port 5 with the velocity vectors indicated by the red solid arrows. d2) The calculated velocity for the dynamic transport along the navy dashed line in d1. d3) The calculated output velocity as a function of the transport distance *l* under various rotation frequency.

A schematic illustration of the proposed RBC microrouter is shown in **Figure** **1**a. Under optical manipulation, five RBCs are trapped one by one and then arranged into a regular pentagon shape. By scanning the laser beams in a circular manner, these RBCs will rotate around their own axes under the action of optical torque. Meanwhile, their rotation velocities and directions can be modulated in real time. Each rotating RBC serves as an endogenous cellular microrotor, which will drive the surrounding blood to generate a localized microflow filed. By designing specific rotational mode of RBC microrotors, the induced microflow field can be precisely sculpted in a programmable manner. In this way, an RBC microrouter will be constructed and is expected to realize dynamic transport and selective routing of various targets in vivo.

To interpret the above operation principle more quantitatively and verify its feasibility, the microflow field induced by the RBC microrouter was investigated in silico by finite‐element simulation. The detailed routing procedure through the microflow could be divided into three successive steps: dynamic input, programmable processing, and controlled output. First, a microrouter was assembled based on five RBCs with a uniform rotation frequency of *f* = 1 Hz, and the target (a particle with the radius of 1 µm) was located outside near the RBC 3 and 4 (Figure 1b1). Four RBCs (RBC 1, 2, 4, and 5) were rotated in a clockwise direction (red dashed arrows) while RBC 3 was rotated in an anticlockwise direction (blue dashed arrow). Consequently, the microflow surrounding the target moved along the +*y* direction (navy arrows in Figure 1b1, Movie S1, Supporting Information). Within the range of 0 µm < *y* < 8 µm, the flow velocity (*V*) was mainly attributed to the vertical component (*V_y_*) while the horizontal component (*V_x_*) fluctuated around zero (region I in Figure 1b2). In this range, the target would be directed into the microrouter by the microflow, thus realizing dynamic input. The value of *V* increased gradually and then reached a maximum velocity of *V_max_* = 12.5 µm s^−1^, where it arrived at the center of RBC 3 and 4 (i.e., *y* = 8 µm). Meanwhile, *V_max_* exhibited a linear relationship with *f* (Figure 1b3), indicating that the target input could be accelerated by increasing the rotation speed of the RBC microrouter.

After target input, *V* started to coincide with *V_x_* for the range of *y* > 12.5 µm (region II in Figure 1b2), where the target would move along the +*x* direction instead of +*y* direction. Subsequently, all the RBC microrotors were programmed to rotate in the clockwise direction. As a result, the inner fluid started to flow around the center of microrouter (e.g., the blue dashed circle in Figure 1c1). In this trajectory, *V_x_* and *V_y_* exhibited a sinusoidal distribution with a phase difference of π/2, while the resultant velocity *V* remained constant (Figure 1c2). Consequently, the target would experience a synchronous rotation (Movie S1, Supporting Information), for which *V* could be modulated by regulating the rotation radius *r* and the rotation frequency *f* of RBC microrotors (Figure 1c3). Benefited from such inner rotation, the target could pass through five ports sequentially, making it possible for selective output at the desired port.

Once the target reached a specific port (such as port 5), the rotation of the nearby RBC 1 was switched to the anticlockwise direction to induce a directional microflow for the controlled output from port 5 (Figure 1d1). With the increase of transport distance *l*, the flow velocity *V* was increased first and then decreased later (region I&II in Figure 1d2), with a maximum value achieved at the the center of RBC 1 and 5 (i.e., *l* = 5.5 µm). Importantly, the direction of the actuation flow remained relatively stable at ≈*θ* = 54°, thus ensuring the output of the target from port 5 (Figure 1d2, Movie S1, Supporting Information). Furthermore, the output velocity could be elevated by increasing the rotation frequency *f* (Figure 1d3). Thus, by combing the functions of dynamic input, programmable processing and controlled output, a real‐time routing of the target along an exclusive trajectory can be achieved. It is worth noting that five RBC microrotors were required to assemble the microrouter, with the aim to break the inherent symmetry for achieving independent and simultaneous control of multiple targets.^[^ ^30^ ^]^

### 2.2 Flexible Assembly of RBC Microrouter in Vivo

**Figure 2:**
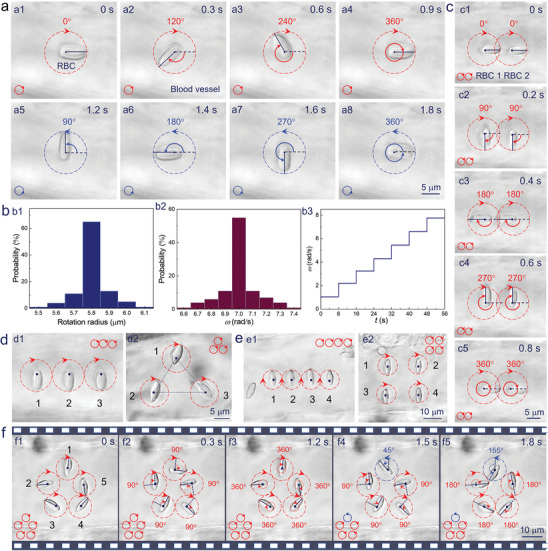
a) Optical microscopic images for rotating one RBC in a clockwise a1–a4) and anticlockwise a5–a8) direction. b) The calculated probability distribution for the rotation radius b1) and angular velocity b2). b3) The calculated angular velocity as a function of time. c) Optical microscopic images for rotating two RBC microrotors in a clockwise direction with a period of 0.8 s. d) Optical microscopic images for arranging three RBC microrotors into a line or triangle shape with a clockwise rotation. e) Optical microscopic images for arranging four RBC microrotors into a line or rectangular shape with a clockwise rotation. f) Optical microscopic images for assembling the RBC microrouter with an adjustable rotation velocity and direction.

Based on the above simulation results, the assembly of RBC microrouter in the blood vessel of living zebrafish was explored. We first characterized the rotation flexibility of the optically trapped RBCs. As shown in **Figure** **2**a, one RBC was trapped in the blood vessel due to the fixed optical potential well (OPW), which was set by specifying the illumination location of laser beam focus on the RBC (navy dot). Meanwhile, a collection of illuminated points was designed to introduce a circularly scanning pattern for the laser beam (red dashed circle), under which the RBC started to rotate in a clockwise direction with a rotation period of 0.9 s. By inverting the scanning direction at *t* = 1.2 s, the RBC began to rotate in the anticlockwise direction with the same rotation period (Figure 2a and Movie S2, Supporting Information). This controlled rotation exhibited a high stability, with an average rotation radius and velocity of 5.8 µm and 7.0 rad s^‐1^, respectively (Figure 2b1&2). Moreover, the rotation velocity *ω* could be regulated in real time (1.0 to 8.0 rad s^−1^) by regulating the switching rate of acoustic‐optic deflector (AOD) and the number of illuminated points in the circular sequence (Figure 2b3; Figure S1, Supporting Information).

Then, the simultaneous rotation of multiple RBCs was investigated. As indicated in Figure 2c, two RBC microrotors were trapped by placing two fixed OPWs (navy dots) and actuated by the circularly scanning laser beams as mentioned above. As a result, they experienced a synchronous rotation in the clockwise direction with a rotation period of 0.8 s (Movie S2, Supporting Information). Similarly, three or four RBCs could also be arranged into a designed pattern and rotated simultaneously (Figure 2d&e). Finally, the desired RBC microrouter was assembled in the blood vessel by arranging five RBCs into a pentagon shape (cell distance: 10–18 µm) and rotating them in the clockwise direction with a period of 1.2 s (Figure 2f1–3). During the assembly and routing processes, five RBCs were located at the same height level in the Z‐axis direction, which is attributed to the action of optical gradient force (Figure S2, Supporting Information). Importantly, by regulating the scanning direction and speed of the laser beam on the RBCs, each one of them (e.g., RBC 1) could be controlled to rotate in an opposite direction or with a different velocity while the others remained unchanged (Figure 2f4&5, Movie S2, Supporting Information). Therefore, an independent control could be achieved for the individual biological building block in the RBC microrouter, with the aim to sculpture the microflow field and achieve multifunctional routing in a precise and programmable manner.

### 2.3 Performance Characterization for the RBC Microrouter

**Figure 3:**
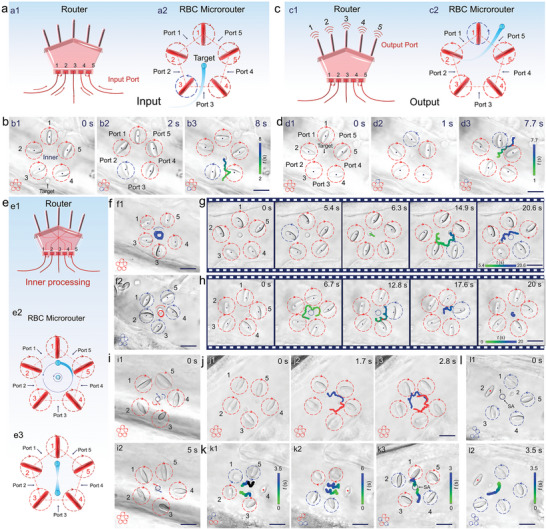
a, c) Schematic illustration for modulating the RBC microrouter to import a2) or export c2) a target, which is similar to a macroscopic router for the signal input a1) or output c1). b,d) Optical microscopic images for importing (b) or exporting (d) one target from port 3 and 5 of the assembled RBC microrouter, respectively. The motion of the target with time is demonstrated from green to blue along the trajectories. e) Schematic illustration for the controlled rotation or linear transportation of the targets inside the microrouter, just like the inner signal processing of a macroscopic router. f) Optical microscopic images for rotating the target inside the microrouter in the anticlockwise f1) or clockwise f2) direction. g) Optical microscopic images for rotating the inner target with an increased radius. h) Optical microscopic images for rotating the inner target followed by a directional transport toward the center. i,j) Optical microscopic images for (i) a simultaneous rotation of two targets in the center of the microrouter, followed by (j) a controlled separation through the increase of rotation radius. k) Optical microscopic images for transporting two targets toward the right k1), left k2) and two opposite directions k3), with their detailed motions indicated by the time‐mapping colorful curves. l) Optical microscopic images for rotating one target while keeping the other stationary. Scale bar: 10 µm.

After the assembly of RBC microrouter, its performance for dynamic input, inner processing, and controlled output was characterized. **Figure** **3**a&c shows the schematic illustration for the assembled RBC microrouter, which can be regarded as a multi‐path router with five input ports and output ports. According to the simulation result (Figure 1b&d), it is expected that the assembled microrouter can import or export a target through a specific port by switching the rotation direction of a corresponding RBC microrotor (Figure 3a2&c2). To verify this hypothesis, a cell nucleus (extracted from RBC, radius: 1 µm) was optically trapped as a biological target and placed outside of the port 3 of the assembled microrouter (Figure 3b1). At *t* = 2 s, the RBC microrotor 3 was regulated to rotate in the anticlockwise direction (Figure 3b2). As a result, the target was imported to the microrouter via port 3 successfully (Figure 3b3). In addition, two targets could also be input simultaneously from different ports (Figure S3, Supporting Information). Furthermore, by switching the rotation direction of RBC microrotor 1 to the anticlockwise direction, the target could be exported from the microrouter through port 5 (Figure 3d, Movie S3, Supporting Information). By using this method, five targets could be imported or exported from five different ports in a programmable manner (Figures S4 and S5, Movie S3, Supporting Information). These results indicate that the assembled RBC microrouter in the blood vessel can effectively realize the target input and output functions as designed.

After importing the target, the assembled RBC microrouter exhibited a powerful inner processing function, under which the targets could experience a controlled rotation, linear transport and designed separation, just like an intelligent macroscopic router that can execute complex signal processing (Figure 3e). In accordance with the simulation result (Figure 1c), by rotating all the RBC microrotors in the same direction, the imported target started to rotate inside the microrouter and the rotation direction could be switched by reversing the microrotors (Figure 3f, Movie S4, Supporting Information). Moreover, the rotation radius of the target could be modulated in real time. As shown in Figure 3g, a linear transport, indicated by the green curve, was introduced for a rotating target toward the outer of the microrouter by reversing the rotation direction of RBC microrotor 2 at *t* = 5.4 s. After that, the target was rotated again in a larger radius, i.e., from 0 to 6 µm. In a similar way, by transporting the target toward the center of the microrouter, the rotation radius could be decreased in a controlled manner, e.g., from 5 to 0 µm (Figure 3h).

The assembled RBC microrouter could also be exploited to process two targets simultaneously. As shown in Figure 3i, two targets were located closely at the center of the microrouter and rotated together (indicated by the blue and red arrows, Movie S4, Supporting Information). By increasing their rotation radius to 7 µm using a similar method as mentioned above, the two targets were clearly separated (Figure 3j, Movie S4, Supporting Information). Furthermore, by rotating RBC 2&3 clockwise and RBC 1&5 anticlockwise while keeping RBC 4 still, two targets could be transported together toward the right (Figure 3k1; Figure S6, Supporting Information), and the transport direction could be reversed by switching the rotation direction of the four RBC microrotors (Figure 3k2; Figure S6, Supporting Information). Interestingly, when we rotated RBC 1, 3&5 clockwise and RBC 2&4 anticlockwise, a stagnation area (SA), where the flow velocity was zero (Figure S7, Supporting Information), was created for the inner microflow field of the microrouter (indicated by the navy circle). From this area, two targets could be transported in the opposite directions (Figure 3k3, Movie S4, Supporting Information). Alternatively, by keeping RBC 2 still and rotating other four RBCs anticlockwise, the target 1 could remain stationary at the SA, while the target 2 was driven to rotate inside the microrouter (Figure 3l; Figure S8, Movie S4, Supporting Information). Furthermore, the proposed RBC microrouter could be exploited to process the larger biological cells, such as the platelets and WBCs (Figures S9–S11, Supporting Information). These results demonstrate the dynamic inner transportation and separation abilities of the assembled microrouter, owing to the programmable regulation of the five RBC microrotors.

**Figure 4:**
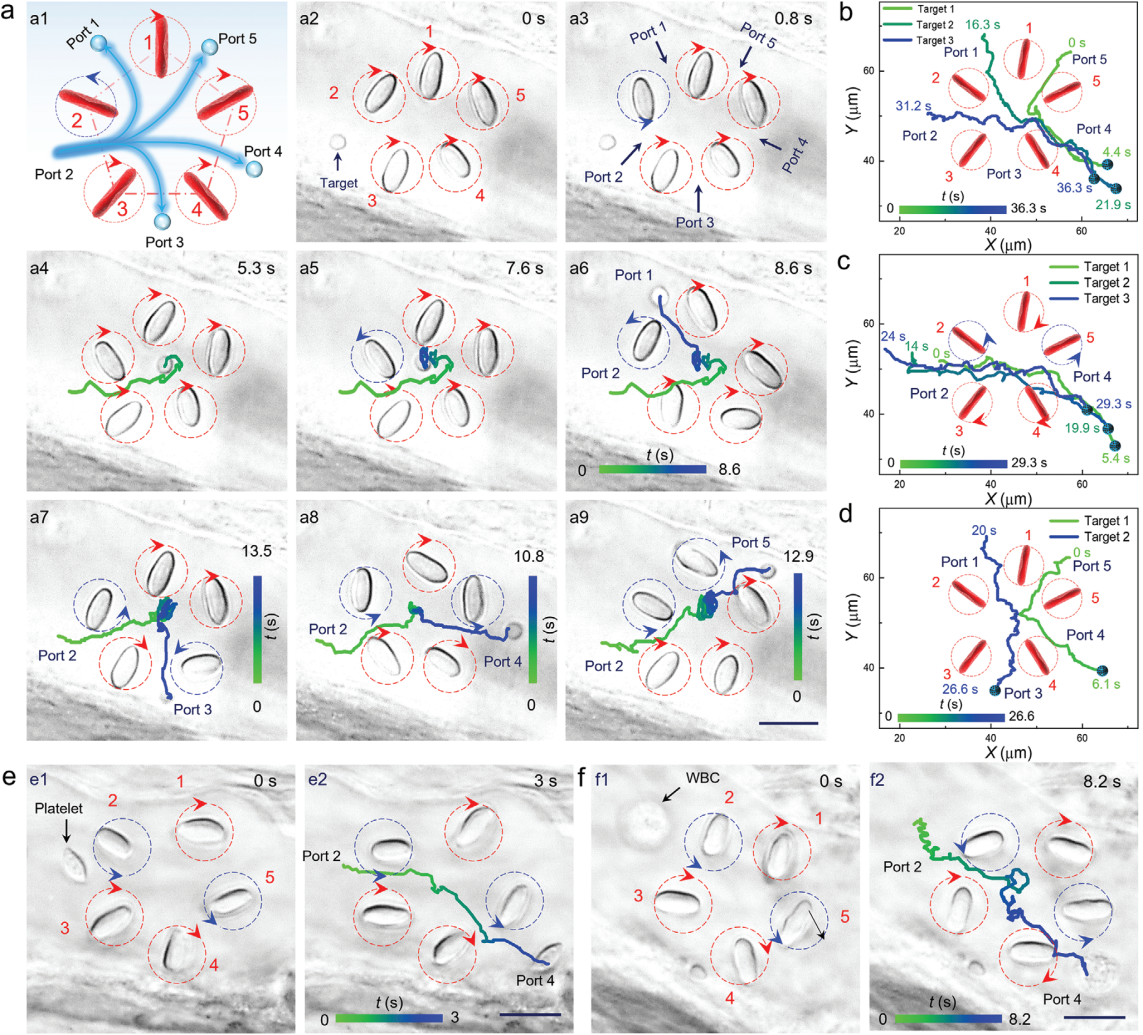
a) Schematic illustration a1) and optical microscopic images for using RBC microrouter to import the target from port 2 a2–5) and then export from port 1 a6), 3 a7), 4 a8), and 5 a9), respectively. b) Optical routing for importing three targets from various ports (port 5, 1, 2) while exporting them from the same port (port 4). c) Optical routing for importing three targets from the same port (port 2) and then exporting them from port 4. d) Optical routing for importing and exporting two targets in different ports. e, f) Optical microscopic images for routing platelet (e) and WBC (f) with the detailed trajectory indicated by the time‐mapping colorful curves. Scale bar: 10 µm.

Based on its programmable inner processing ability, the microrouter was further tested for diversified input and output of various targets. The input of the target from a specific port while output through different ports were first demonstrated (**Figure** **4**a1). As shown in Figure 4a2–4, a target (cell nucleus) at the entrance of port 2 was imported into the microrouter by rotating RBC 2 anticlockwise. After that, the rotation of RBC 2 was switched to the clockwise direction at *t* = 5.3 s (Figure 4a4). Thus, the target started to rotate around the center of microrouter (Figure 4a5) and was transported to port 1 at *t* = 7.6 s. At this moment, RBC 2 was rotated anticlockwise, resulting in the successful output of the target from port 1 (Figure 4a6). Similarly, the target imported from the same port (i.e., port 2) could also be transported to port 3, 4 or 5 inside the microrouter via linear transportation or rotation around the center, followed by output from the corresponding port through switching the rotation direction of RBC 4, 5 or 1, respectively (Figure 4a7–9 and Movie S5, Supporting Information). Besides, multiple targets could be imported from different ports and then exported from a same port. As shown in Figure 4b, target 1, 2, and 3 were imported from port 5, 1, and 2, respectively. Within the microrouter, all the three targets were delivered to port 4 by linear transportation and then exported from this port by switching the rotation direction of RBC 5 (Movie S6, Supporting Information).

Furthermore, multiple targets could be imported and then exported through the same route. As shown in Figure 4c, by using a successive procedure of switching the rotation direction of RBC 2, linear transportation inside the microrouter and switching the rotation direction of RBC 5, three targets were imported from port 2 and then exported through port 4 along a similar trajectory (Movie S6, Supporting Information). The required routing time was calculated to be 5.4, 5.9, and 5.3 s for the target 1, 2, and 3, respectively. Thus, the average routing rate of the targets was determined to be 10 µm s^−1^, which corresponds to the average intensity of the induced microflow field. Alternatively, the targets could also be imported and exported through independent transport routes (Figure 4d and Movie S6, Supporting Information). For example, target 1 was imported from port 1, experienced a linear transport across the center of the microrouter, and finally got exported through port 3. While target 2 entered the microrouter from port 5 and was subsequently exported from the nearby port 4.

In addition to the cell nucleus, the routing capability was also characterized for larger blood cells, including the platelet and WBC. As indicated in Figure 4e, by rotating RBC 2&5 anticlockwise and 1, 3&4 clockwise, one platelet was imported from port 2 and then exported from port 4, with a transport distance of 45 µm in 3 s. By using the same method, one WBC (diameter: 8.5 µm) was also transported through a similar route (Figure 4f). Moreover, the platelet and cell nucleus could be selectively transported into two branches, thus achieving a controlled separation for various targets (Figure S12, Supporting Information). Therefore, the assembled microrouter has flexible routing capacity for various biological targets.

### 2.4 Biomedical Applications of the RBC Microrouter

**Figure 5:**
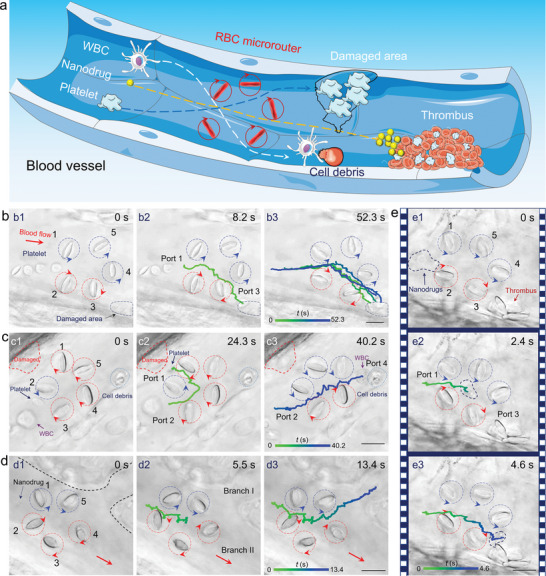
a) Schematic illustration for directing platelets for controlled hemostasis, transporting WBCs for targeted clearance, and active delivery of antithrombotic nanodrugs with RBC microrouter. b) Optical microscopic images for routing four platelets toward the damaged vessel. c) Optical microscopic images for transporting the platelet and WBC toward the damaged vessel and cell debris, respectively. d) Optical microscopic images for routing the nanodrug into branch I. e) Optical microscopic images for routing a swarm of nanodrugs toward the thrombus. The detailed transport trajectory was indicated by the time‐mapping colorful curve. Scale bar: 10 µm.

Based on the above functions, the assembled microrouter was applied for a series of biomedical applications, such as directing platelets for controlled hemostasis, transporting WBCs for targeted clearance, and active delivery of antithrombotic nanodrugs (**Figure** **5**a). First, the continuous routing of four platelets was performed toward the damaged vessel for a desired hemostasis. Considering that the spontaneous approaching of platelets would require an uncertain time due to the low amount and stochastic distribution of platelets in blood vessels, four flowing platelets were trapped first and then arranged into a line in front of the assembled RBC microrouter to evaluate its continuous routing capability (Figure 5b1). In order to guide these platelets to the damaged vessel wall, the port 1 of the microrouter was designed to be in front of the platelets, while port 3 faced the damaged area. By rotating RBC 2&3 clockwise and RBC 1, 4&5 anticlockwise, the platelets were imported from port 2 sequentially and then exported through port 3 to arrive at the damaged vessel wall (Movie S7, Supporting Information).

Considering that the RBC microrouter has the target separation ability, we applied it for transporting different targets to execute different biological tasks. As shown in Figure 5c1, a platelet and a WBC were located in the center of the blood vessel, and there was a damaged area and cell debris at the left and right side of the blood vessel, respectively. An RBC microrouter was assembled and programmed to import the two targets from port 2, followed by exporting the platelet through port 1 and the WBC through port 4, respectively. In this way, the platelet was directed toward the damaged vessel for hemostasis (Figure 5c2), while the WBC was navigated to the cell debris for a targeted clearance by phagocytosis (Figure 5c3, Movie S8, Supporting Information).

Since the microrouter can induce a microflow to actuate the movement of various targets in a certain direction, we further explored its potential for active delivery of nanodrugs. For the desired antithrombosis treatment, mesoporous silica nanoparticles (MSNs) were chosen as the carrier for loading the antithrombotic drug urokinase due to their negatively charged surface potential, uniform mesopores, and large surface area (Figure S13, Supporting Information)^[^ ^31^, ^32^, ^33^ ^]^ After the preparation, the nanodrugs were introduced into the blood vessel through a microinjection method. At *t* = 0 s, a nanodrug was flowing in the main blood vessel, and there was a branch on its left (Figure 5d1). Then an RBC microrouter was assembled in front of it. By reversing the rotation direction of RBC 1&5, the MSN was successfully transported into the branch at *t* = 13.4 s (Figure 5d3 and Movie S9, Supporting Information). Because the motion of the nanodrug was driven by the microflow, the quantity of the MSNs that could be transported by the microrouter should not be limited to one. To verify this assumption, an RBC microrouter was placed between the thrombus and a swarm of nanodrugs (Figure 5e1). After reversing the rotation direction of RBC 1, 4&5, the nanodrugs were imported from port 1 and exported through port 3 to the thrombus on the vessel wall, thus realizing the targeted delivery of nanodrugs in a large quantity (Figure 5e3 and Movie S9, Supporting Information).

## 3 Discussion

In this work, a living microrouter was demonstrated by integrating the endogenous RBCs, programmable SOTs and flexible optofluidic manipulation, thus constructing a multifunctional biomedical platform to achieve diversified routing for various targets toward specific destinations in vivo. With the assistance of the RBC microrouter, various blood cells, such as platelets and WBCs, were transported toward the damaged vessel and cell debris for the dynamic hemostasis and targeted clearance, respectively. Moreover, the microrouter also enables a precise transport of nanodrugs in a large quantity, which might provide a biocompatible tool for active and targeted drug delivery in the treatment of thrombosis.

In addition, the proposed RBC microrouter exhibited a high assembly stability and rotation flexibility during the continuous rotation without a significant degradation (Figure S14, Supporting Information), thus providing a great potential for its long‐term in vivo applications. The microrouter consisted of five different RBCs still has the capacity for a desired routing of targets in vivo (Figure S15, Supporting Information). The rotation flexibility of the RBC microrouter might be affected by the plasma viscosity and flow velocity. A larger viscosity and a faster blood flow will require an enhanced optical force to ensure the flexible rotation of the RBC microrouter. Meanwhile, a fast blood flow will provide a shorter reaction time for the desired recognition of targets and programming modulation of RBC microrouter. In the presented work, the plasma exhibited a viscosity of ≈1.2 × 10^−3^ Pa s and all the experiments were performed under the flow velocity smaller than 30 µm s^−1^. Moreover, the major targets are significantly different in morphology and size, which can be easily recognized under a white light camera. For future study, if we need to investigate other targets with similar morphology and size, e.g., neutrophils, eosinophils and basophils, fluorescence labelling can be used to differentiate them from each other. Besides, the ideal depth was estimated to be within 500 µm, under which the targets will be visualized clearly and the RBC microrouter could be assembled for a desired routing. Furthermore, various routing programs could be summerized into a instruction library. Thus, for the unknown target location due to natural blood flow in vivo, a desired routing could be achieved by directly calling the corresponding program on the RBC microrouter in real time. By integrating an intelligent image recognition and closed‐loop feedback control, the target position could be monitored in real time and then the desired routing program will be implented in a fully automatic manner at video rates (maximum: 60 *f* s^−1^), thus achieving a precise routing with a higher flexibility and efficiency.

The existing microrouting techniques include the hydrodynamic strategy, magnetic tweezer strategy, optical tweezer strategy, and optically actuated hydrodynamic manipulation strategy. By integrating the nonlinear feedback control^[^ ^34^ ^]^ or trainable artificial neural network,^[^ ^35^ ^]^ the hydrodynamic strategy could achieve the diversified routing while impose no specific constraints on the targets’ physicochemical property and minimize the possible damages to the biological specimens. However, they are usually operated at a large scale, and challenging to be implanted in vivo due to the required connection with microfluidic devices. The magnetic tweezer can achieve a selective routing in a noncontact manner with a large operation depth in vivo.^[^ ^36^ ^]^ However, it is limited to the specific magnetic targets and requires an elaborate decoration for the biospecimen.^[^ ^23^ ^]^ With the high spatio‐temporal precision and remote activation capability, optical tweezers can route various targets directly in a real‐time programming manner.^[^ ^37^, ^38^ ^]^ Nevertheless, a direct irradiation will exert on the targets and many types of absorbing particles cannot be optically routed. To address this issue, Phillips et al. constructed an excellent optically actuated hydrodynamic manipulation system, under which various targets could be manipulated in a dynamically reconfigurable manner without directly illuminating them.^[^ ^30^ ^]^ However, the reported work mainly exhibited the motion and direction control for the targets in vitro, rather than focusing on the precise routing of various targets in vivo. Meanwhile, an invasive implantation and elaborate surface decoration will be required to introduce the exogenous rotors into the blood vessel and ensure their biocompatibility in vivo. In this work, the proposed strategy for making a programmable medical micromachine (PMM) is fully based on the intracorporal biological cells and combines the intrinsic advantages of scanning optical tweezers and optofluidic manipulation technique. By exploiting the endogenous RBCs, the obtained microrouter avoids the invasive implantation of exogenous synthetic materials, leading to high biocompatibility without any invasive tissue damage or unwanted immune response. Besides, through the dynamic scanning of beam focus in a time‐sharing manner, the RBC microrouter can be constructed at designated site by arranging and rotating five RBCs in an independent and programmable manner, thus achieving a desired microflow field with adjustable velocity and direction. Furthermore, the optofluidic strategy, based on the actuation by the induced microflow, was utilized to enable the flexible routing of various targets in a single‐cell precision and programmable manner. This strategy does not require direct laser illumination on the targets and thus is free from strict restrictions for the intrinsic material properties of the targets. Additionally, a highly localized actuation microflow was achieved by the microrouter for a desired near‐field routing without affecting the surrounding cells, thus avoiding the indiscriminate delivery of all immersed targets by the large‐scale flow fields.

The possible biological damages were carefully considered for this optically programmable RBC microrouter. Beneficial from the optofluidic manipulation, the laser beam and the targets (i.e., biological cells or nanodrugs) are physically separated. Only the five RBCs were directly irradiated by beam focus for optical trapping and controlled rotation. To minimize possible damages on them, a laser beam at the wavelength of 1064 nm was chosen to construct the RBC microrouter due to the relatively low photon absorption at this wavelength. The maximum laser power used in this study was 200 mW, which might induce a temperature increase of 1.6 °C in vitro.^[^ ^39^, ^40^ ^]^ Furthermore, the potential cell damage was examined for the optically manipulated RBCs by acridine orange/ethidium bromide (AO/EB) staining. The results demonstrated there was no observable cell injury (Figure S16, Supporting Information) or tissue damage (Figure S17, Supporting Information) during the dynamic routing. That might be attributed to the weaker light intensity that arrived at the targeted RBCs in vivo, because of the unavoidable scattering by the skin and tissue. In addition, the generated heat could be conducted in a rapid and efficient manner because of the high thermal conductivity of the blood flow, thus preventing the potential heat accumulation.

There are still some issues to be addressed for applying this strategy more extensively. First, it might face challenges to achieve a clinical translation due to the limited penetration depth of light. Fortunately, the manipulation depth has been pushed forward for OTs with the assistance of optical coherence compensation technology,^[^ ^41^ ^]^ adaptive optical microscopy,^[^ ^42^ ^]^ reflection matrix optical coherence tomography^[^ ^43^ ^]^ and guidestar‐assisted wavefront‐shaping methods.^[^ ^44^ ^]^ Furthermore, by integrating fiber tweezer with optical fiber endoscopic device, the focused laser might be introduced inside the body to manipulate RBCs in internal tissues.^[^ ^45^ ^]^ Second, achieving a tight focus using a high numerical‐aperture objective (i.e., NA = 1) was technically demanding due to limitations imposed by tissue scattering and aberrations. However, it could be improved by integrating the in situ wavefront correction technique, which can allow a desired compensation of all aberrations along the entire optical train.^[^ ^46^ ^]^ Meanwhile, benefited from the optimized focusing on a larger depth, the required laser power will be further decreased, thus providing a great potential to manipulate RBCs in the human body at a lower laser power. Thrid, an intelligent image recognition and closed‐loop feedback control can be further introduced to monitor the target position and guide the regulation of specific RBC microrotors, respectively.^[^ ^8^, ^30^ ^]^ Consequently, the routing procedure will be fully handed over to the designed iterative algorithm, thus being beyond the manual control and achieving millisecond modulation with nanoscale finesse.^[^ ^47^, ^48^ ^]^ Besides, the diameter of blood vessel should be larger than 30 µm to allow the dynamic construction of pentagon‐shaped RBC microrouter. Thus, it might be challenging to assemble the RBC microrouter in the capillary with narrow diameter. Fortunately, some relatively simple routing functions can be achieved by using RBCs with less numbers or microrotors with smaller size (e.g., cell nucleus) .^[^ ^26^, ^29^ ^]^ Finally, the routing capability might be affected by the spontaneous blood flow. Thus, as a first step toward the disease treatment, the assembled microrouter can be exploited in an occlusive blood vessel or inside a large tumor where the influence of blood flow is minimized. For example, anti‐thrombolytic drugs can be routed to ameliorate thrombosis. In addition, specific WBCs such as M1 macrophages and T lymphocytes can be selectively routed toward the tumor tissues for optimized tumor therapy. Besides, the actuation flow could be modulated precisely according to the specific original blood flow, thus achieving the desired microflow distribution through an organic integration of intelligent closed‐loop program.^[^ ^49^ ^]^ Meanwhile, the blood flow is beneficial for the continuous input of targets into the RBC microrouter, which might further improve its routing efficiency.

## 4 Conclusion

In conclusion, a fully biological PMM, i.e., a living microrouter, was designed and constructed from endogenous RBCs for the desired routing of various biological targets toward their respective destinations in vivo. Unlike traditional PMMs, the innovative introduction of endogenous RBCs as biocompatible materials avoided the sophisticated micro/nano‐fabrication of artificial building blocks as well as the following invasive implantation procedure. The presence of programmable SOTs and flexible optofluidic strategy enabled the powerful reconfiguration and near‐field hydrodynamic control, independent of cargos’ composition and minimizing the damage to biological specimens. The flexible routing capacities of the microrouter, including dynamic input, programmable inner processing, and controlled output, were investigated by in silico analysis and characterized in the blood vessel of living zebrafish. On these bases, the microrouter was applied for various potential biomedical applications, such as directing platelets for hemostasis, navigating WBCs for targeted clearance, and active delivery of antithrombotic nanodrugs. It is believed that the proposed RBC microrouter may provide an intelligent, multifunctional, and programmable medical platform for cell separation, drug delivery and immunotherapy.

## 5 Experimental Section

### Experiment Setup

The experiment setup was constructed around a scanning optical tweezer (SOT) system (Tweez250si, Aresis Co., Ltd., Slovenia) which was incorporated with an inverted optical microscope for the experiment observation in real time. The trapping laser beam was a continuous gaussian beam at the wavelength of 1064 nm, which was within the biological window to achieve a large penetration depth. First, the laser beam was interacted with the AOD (maximum switching rate: 100K Hz) to obtain a programmable spatiotemporal distribution in real time. After that, the laser beam was expanded through a beam expander to overfill the pupil of microscope objective, reflected upward by the dichroic mirror, and then refocused onto the zebrafish tail through a 60× water immersion microscope objective (CFI Apo, NA = 1.0). Meanwhile, by using a halogen light source (D‐LH/LC: 12 V, 100 W), the sample was irradiated by the top illumination light which was focused through a condenser. The intravital manipulation was recorded by a high‐speed charged coupled device (CCD) camera and displayed on the computer screen for real‐time monitoring, image acquisition, and video recording.

### Zebrafish Care and Treatment

The adult zebrafish (90 days old) were purchased from the Nanjing Eze‐Rinka Biotechnology Co., Ltd. (Nanjing, China). According to standard procedures, the zebrafish were fed with live brine shrimps, maintained in a clean tank, and cultured with a 14 h light/10 h dark cycle at 28.5 °C. All experiments were conducted in accordance with the ethical standards by the Laboratory Animal Ethics Committee of Jinan University.

### Numerical Simulation

The actuation flow field was simulated through a finite element method with the fluid flow module (rotating machinery, laminar flow) as well as the non‐slip boundary condition by using COMSOL Multiphysics 5.3a. The average diameters of RBCs were set to be 8.6 µm, which was consistent with the experiments. Meanwhile, the plasma density and dynamic viscosity of the blood were set as 1.025 × 10^3^ kg m^−3^ and 1.2 × 10^−3^ Pa s, respectively.

### Preparation of Nanodrugs

The MSN suspension and FITC‐labeled urokinase (URK) were commercially available (Huge Biotech. Co., Ltd., Shanghai, China). Firstly, URK (5 mg) was added into the MSN suspension (0.1 mg mL^−1^, 5 mL), followed by an ultrasonic treatment to obtain a homogeneous mixture. After that, the mixture was stirred (100 rpm) under dark condition for 24 h for drug loading. Finally, the URK‐loaded MSNs, i.e., the nanodrugs, were centrifuged and washed for three times with PBS.

### Injection of Nanodrugs into the Zebrafish

The nanodrugs were diluted with PBS buffer to a concentration of 8 × 10^6^ particles mL^−1^. After an ultrasonic treatment (4800 rpm, 10 min), the monodisperse solution was loaded into a glass micropipette (Outer diameter: 1.14 mm; Inner diameter: 0.5 mm). After that, the diameter of micropipette tip was stretched to 10 µm, with the aim to enable a precise injection while minimize the invasiveness. The zebrafish were treated with a general anesthesia by placing them in a Petri dish containing tricaine solution (0.3 mg mL^−1^) for 8 min. The tricaine concentration and incubation time were optimized to prevent overdose anesthesia. After that, the fish were moved to a 15 × 50 mm cover glass and then fixed on agarose slices. Meanwhile, the zebrafish remained surrounded by a small amount of fluid to help with the respiration. Finally, 10 nL nanoparticle solution was injected into the posterior cardinal vein of zebrafish by manipulating the micropipette with a programmable nanoliter injector (Nanojet III, Drummond Co., Ltd., USA). The injection procedure was typically finished within 1 min, and the activity of the zebrafish under anesthesia was confirmed by monitoring the circulation of blood cells under the microscope.

### Data Analysis

To achieve the detailed motion trajectory, a high‐speed CCD was introduced to record the experiment process with a maximum frame rate of 60 Hz. After that, the time‐lapse images were exported to the ImageJ software, and meanwhile, the location coordinates for biological targets were calculated by using the manual tracking plugin to perform the point‐to‐point tracks. Furthermore, the detailed motion trajectory was drawn with the assistance of Origin software. All data statistical analyses were performed to use the Origin software (version 2018b, OriginLab Inc., USA). The data number for each group was 5 and numerical data were reported as Mean ± SD.

## Conflict of Interest

The authors declare no conflict of interest.

## Supporting information

Supporting Information

Supplementary Movie S1

Supplementary Movie S2

Supplementary Movie S3

Supplementary Movie S4

Supplementary Movie S5

Supplementary Movie S6

Supplementary Movie S7

Supplementary Movie S8

Supplementary Movie S9

## Data Availability

The data that support the findings of this study are available from the corresponding author upon reasonable request.
