# Supplementary material for: Optically Programmable Living Microrouter in Vivo
Source: Adv Sci (Weinh). 2023 Sep 25;10(32):2304103. doi: 10.1002/advs.202304103 (PMC10646234; doi:10.1002/advs.202304103)
Supplement: Supplementary file 1 — Supporting Information [file ADVS-10-2304103-s010.pdf]

ADVANCED SCIENCE
Open Access

Supporting Information

for *Adv. Sci.*, DOI 10.1002/advs.202304103

Optically Programmable Living Microrouter in Vivo

Xiaoshuai Liu, Huaying Wu, Shuai Wu, Haifeng Qin, Tiange Zhang, Yufeng Lin, Xianchuang Zheng* and Baojun Li**

Supporting Information

Optically programmable living microrouter in vivo

Xiaoshuai Liu, Huaying Wu, Shuai Wu, Haifeng Qin, Tiange Zhang, Yufeng Lin, Xianchuang Zheng,* Baojun Li**

E-mail: lxshuai@jnu.edu.cn(X. L.); xczheng@jnu.edu.cn(X. Z.); baojunli@jnu.edu.cn(B. L.)

Guangdong Provincial Key Laboratory of Nanophotonic Manipulation, Institute of Nanophotonics, Jinan University, Guangzhou, 511443 China

Table of Contents

Figure S1. Schematic illustration for the operation principle of rotating RBC.

Figure S2. Schematic illustration for trapping RBC at the beam focus and the detailed distribution of beam focus in SOTs.

Figure S3. Simultaneous import of two targets from two different ports.

Figure S4. Dynamic input of five targets from their respective ports.

Figure S5. Controlled output of five targets from their respective ports.

Figure S6. Simulated microflow field for transporting two targets towards the right or left.

Figure S7. Simulated microflow field for the dynamic separation of two inner targets.

Figure S8. Simulated microflow field for rotating one target while keeping the other still.

Figure S9. Simultaneous rotation of two platelets inside the RBC microrouter.

Figure S10. Dynamic rotation of one WBC inside the RBC microrouter.

Figure S11. Simultaneous rotation of two WBCs with a controlled separation.

Figure S12. Dynamic routing of the cell nucleus and platelet towards different branches.

Figure S13. SEM image of the mesoporous silica nanoparticles (MSNs).

Figure S14. The quantitative characterization for the rotation stability of RBC microrouter.

Figure S15. Simulated microflow field generated by the RBC microrouter consisted of five different RBCs and the corresponding routing experiment.

Figure S16. The viability characterization of optically manipulated RBCs.

Figure S17. Quantitative characterization of heating damage on the surrounding tissues.

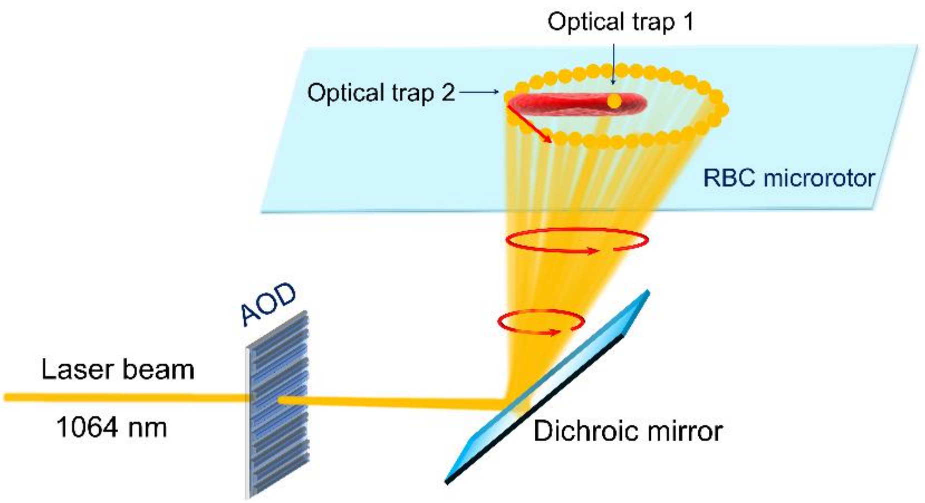

Figure S1. Schematic illustration for the operation principle of rotating RBC. The RBC is trapped by the fixed optical trap 1, while a collection of illuminated points is designed for optical trap 2 to form a circular scanning sequence. Afterwards, the RBC undergoes a synchronous rotation under the action of optical torque, and the rotation velocity can be calculated as follows: $\omega = k_0 f_{AOD}/n$, where k_0 is the proportionality coefficient, f_{AOD} is the switching rate of AOD and n is the number of illuminated points in the designed circular sequence. Therefore, the rotation velocity can be controlled by regulating the switching rate of AOD or the number of illumination points.

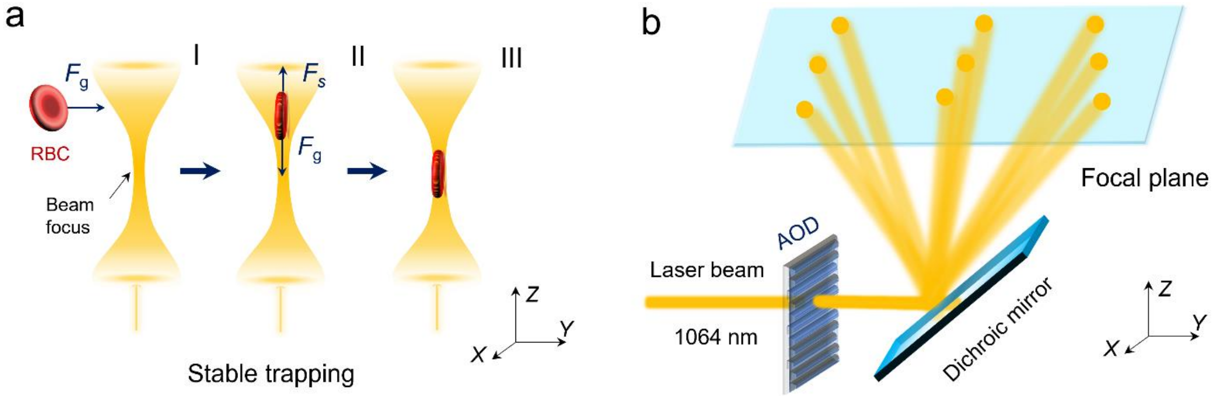

Figure S2. (a) Schematic illustration for trapping the RBC at the beam focus. Once the focused laser beam is irradiated on the RBC, it will generate optical forces including optical scattering force (F_s) and optical gradient force (F_g) , whose directions are along the beam propagation direction and towards the beam focus, respectively. Consequently, the RBC will move towards the optical axis under the action of F_g (stage I), after which F_g will compete with F_s (stage II) to drag the RBC to the beam focus in the Z-axis direction and trap it there (stage III). (**b**) Schematic illustration for the detailed distribution of the beam focus in the scanning optical tweezer system (SOTs). The beam focus is under a programmable scanning and remains at the same height level, *i.e.*, in the focal plane. As a consequence, all the RBCs will be trapped and then rotated at the same height in the Z-axis direction.

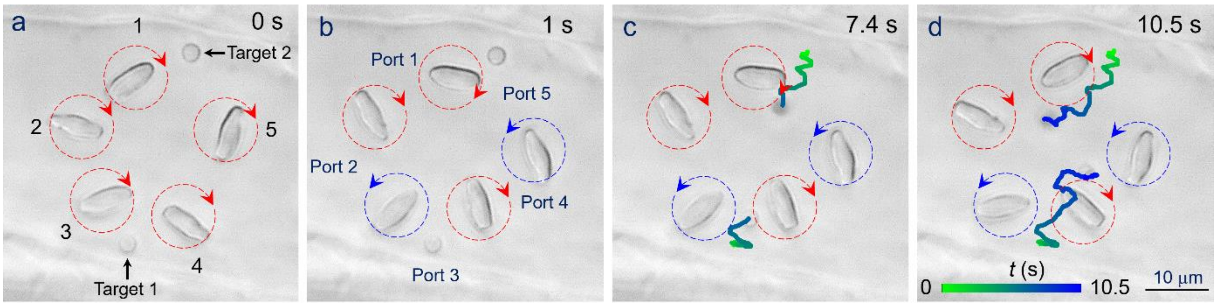

Figure S3. Optical microscopic images for a simultaneous import of two targets from two different ports. (**a**) At $t = 0$ s, two targets, *i.e.*, target 1 and 2, were located at the entrance of port 3 and 5, respectively. (**b**) At $t = 1$ s, the RBC microrotor 3 and 5 were regulated to rotate in the anticlockwise direction. (**c, d**) Consequently, target 1 and 2 were imported into the microrouter in a simultaneous manner via the port 3 and 5, respectively.

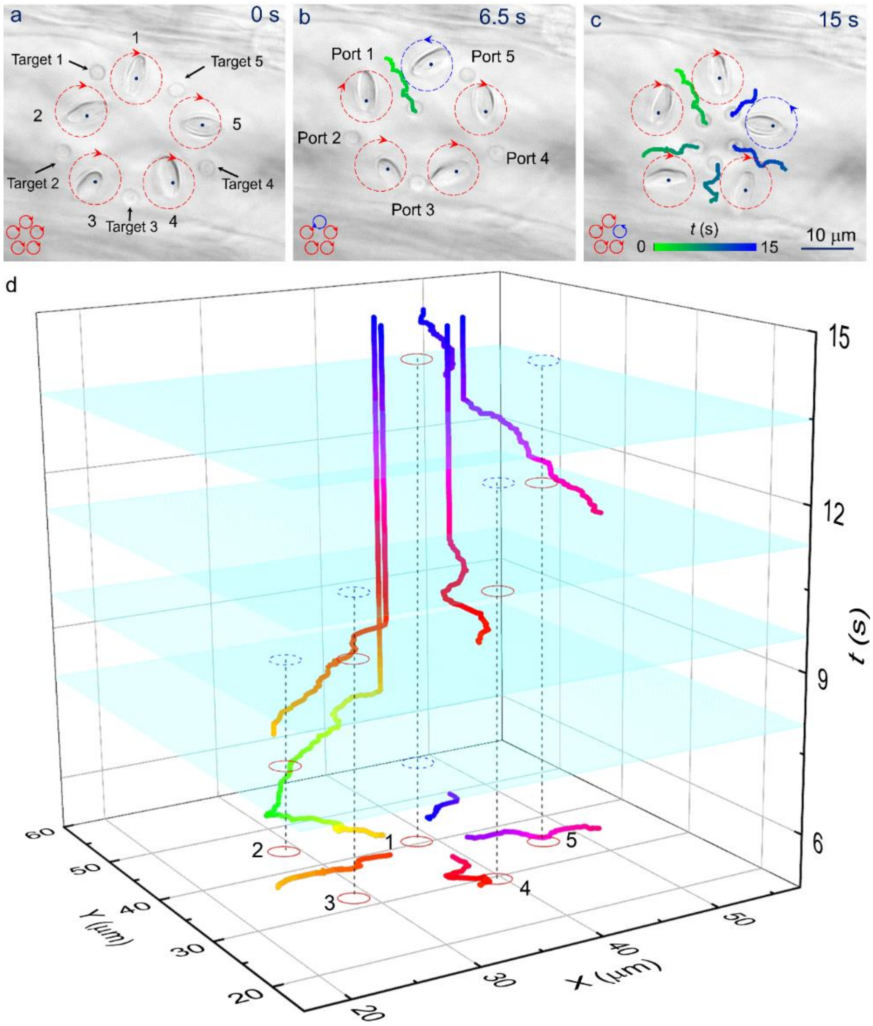

Figure S4. Dynamic input of five targets from their respective ports. (**a**) At $t = 0$ s, five targets (cell nuclei) were located at different ports of the RBC microrouter. (**b**) After RBC 1 was regulated to rotate in the anticlockwise direction, target 1 was imported into the RBC microrouter successfully. (**c**) By regulating the corresponding RBC microrotor in a real-time and programmable manner, the other targets, *i.e.*, target 2, 3, 4 and 5, were also imported from their respective ports one by one. (**d**) The calculated motion trajectory for the five targets as a function of time.

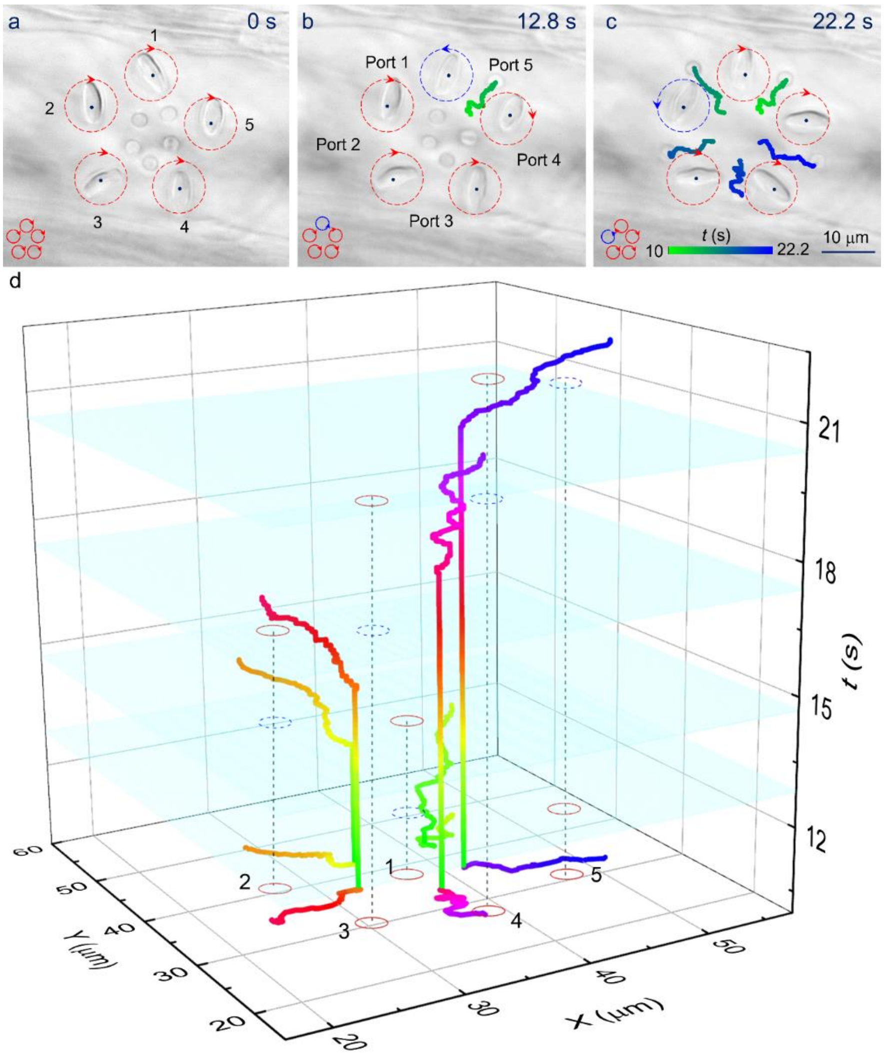

Figure S5. Controlled output of five targets from their respective ports. (**a**) At $t = 0$ s, five targets (cell nuclei) were located near different ports in the inner of the RBC microrouter. (**b**) After the RBC microrotor 1 was regulated to rotate in the anticlockwise direction, target 1 was exported from port 5 successfully. (**c**) By regulating the corresponding RBC microrotor in a real-time and programmable manner, the other targets, *i.e.*, target 2, 3, 4 and 5, were also exported from their respective ports one by one. (**d**) The calculated motion trajectory for the five targets as a function of time.

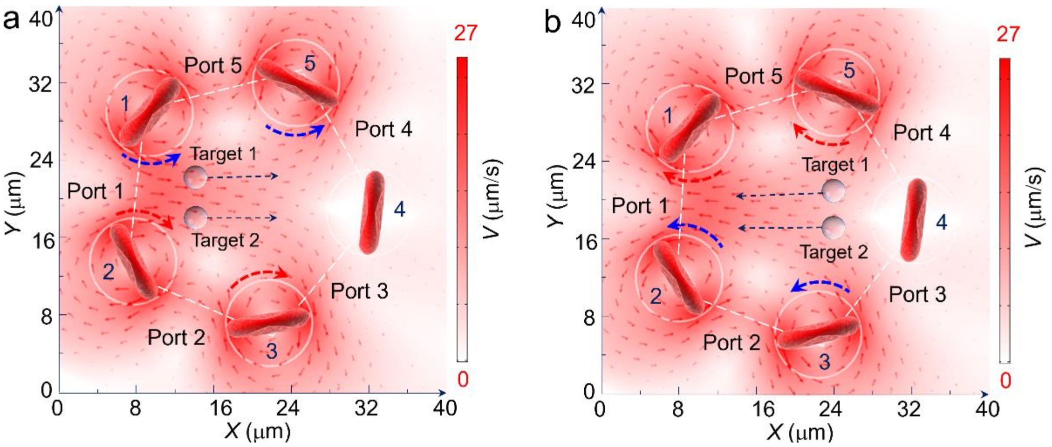

Figure S6. Simulated microflow field for transporting two targets towards the right (a) or left (b).

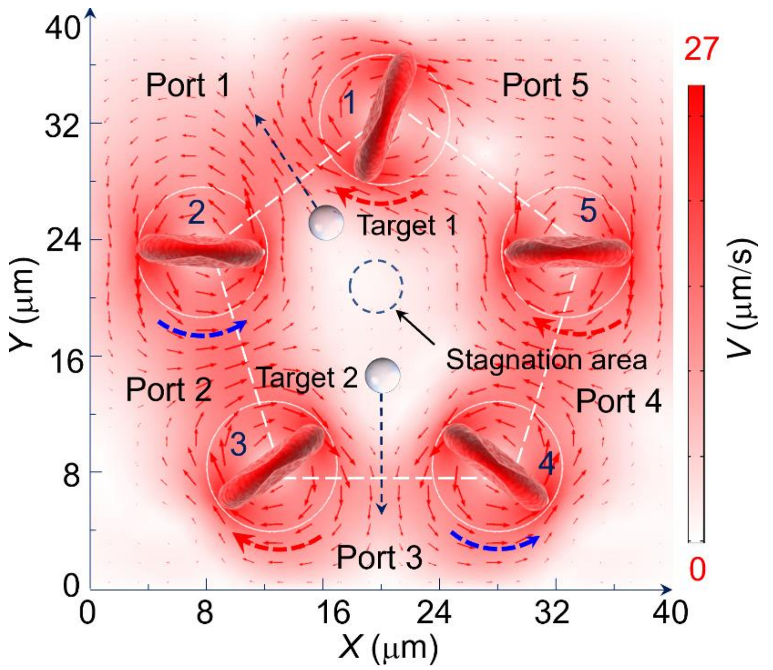

Figure S7. Simulated microflow field for the dynamic separation of two inner targets. By rotating RBC 1, 3 and 5 clockwise and RBC 2&4 anticlockwise, a stagnation area was induced at the center of RBC microrouter, where target 1 and 2 were transported towards port 1 and 3, respectively, thus achieving a dynamic separation.

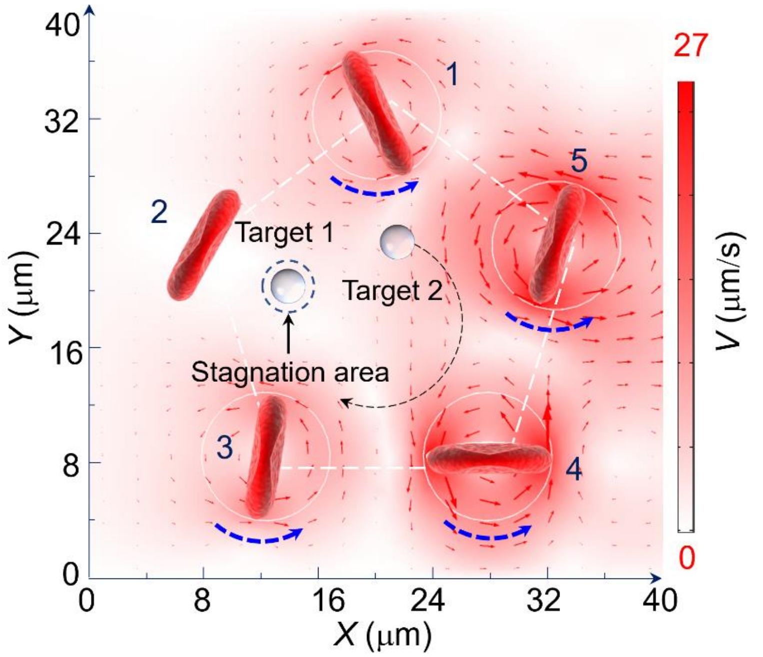

Figure S8. Simulated microflow field for rotating target 2 while keeping target 1 still. By rotating RBC 1, 3, 4 and 5 anticlockwise and fixing RBC 2, target 1 remained stationary at the stagnation area, while target 2 underwent a dynamic rotation along the clockwise direction.

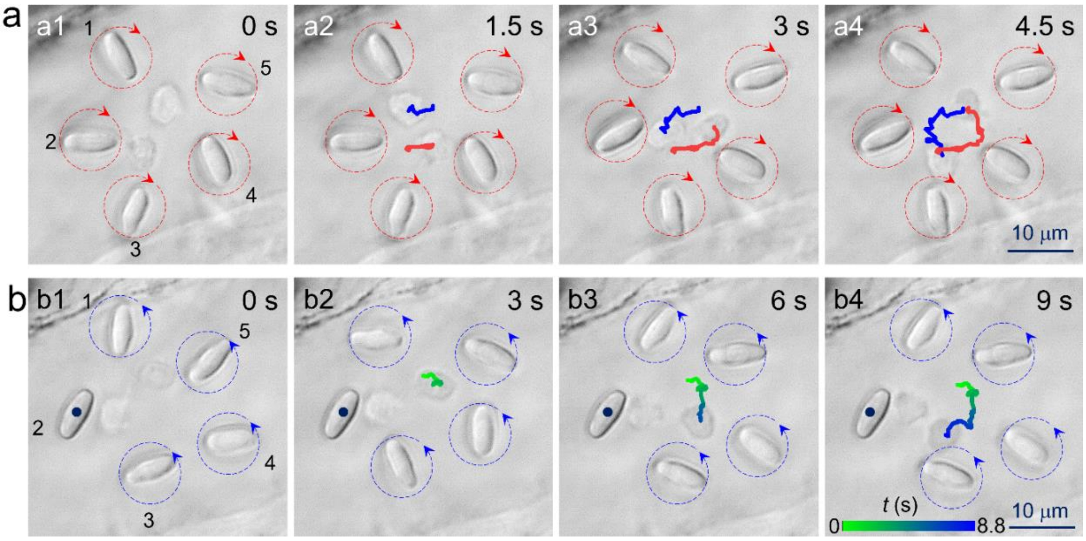

Figure S9. (**a**) Optical microscopic images for a simulatensous rotation of two platelets in the RBC microrouter with a controlled separation. Their detailed trajectories are indicated by the blue and red curves, respectively. (**b**) Optical microscopic images for rotating one platelet while keeping the other stationary.

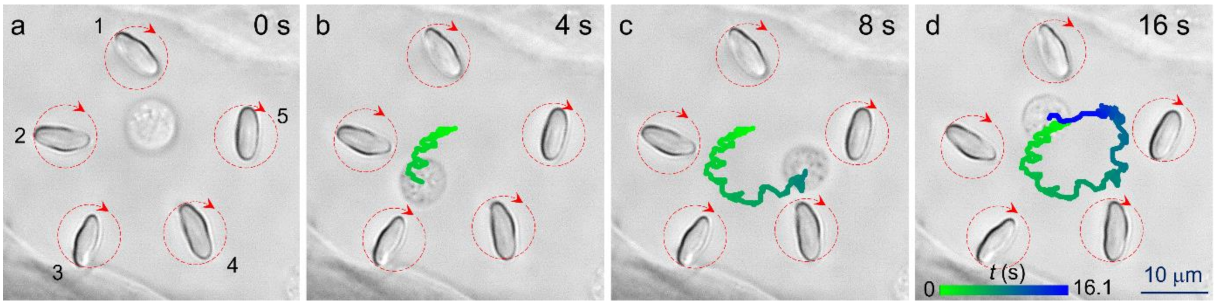

Figure S10. Optical microscopic images for rotating one WBC inside the RBC microrouter. (**a**) At $t = 0$ s, one WBC was located in the inner of RBC microrouter while five RBCs were rotating in a clockwise direction. (**b, c**) The WBC was rotating in a anticlockwise direction and passed through the port 2 (**b**) and 4 (**c**) in a sequential manner. (**d**) At $t = 16.1$ s, the WBC returned to the initial position with a rotation period of 16.1 s. The detailed motion trajectory is indicated by the time-mapping colorful curve.

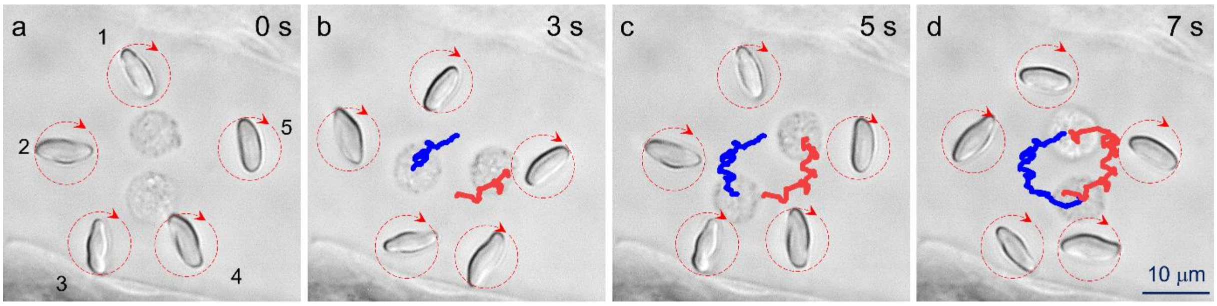

Figure S11. Optical microscopic images for a simultaneous rotation of two WBCs with a controlled separation. (a) At $t = 0$ s, two WBCs were located at the center of RBC microrouter with five RBCs rotating in a clockwise direction. (b-d) The two WBCs underwent a simulatneous rotation in a anticlockwise direction with a designed separation, with the detailed motion trajectory indicated by the blue and red curves, respectively.

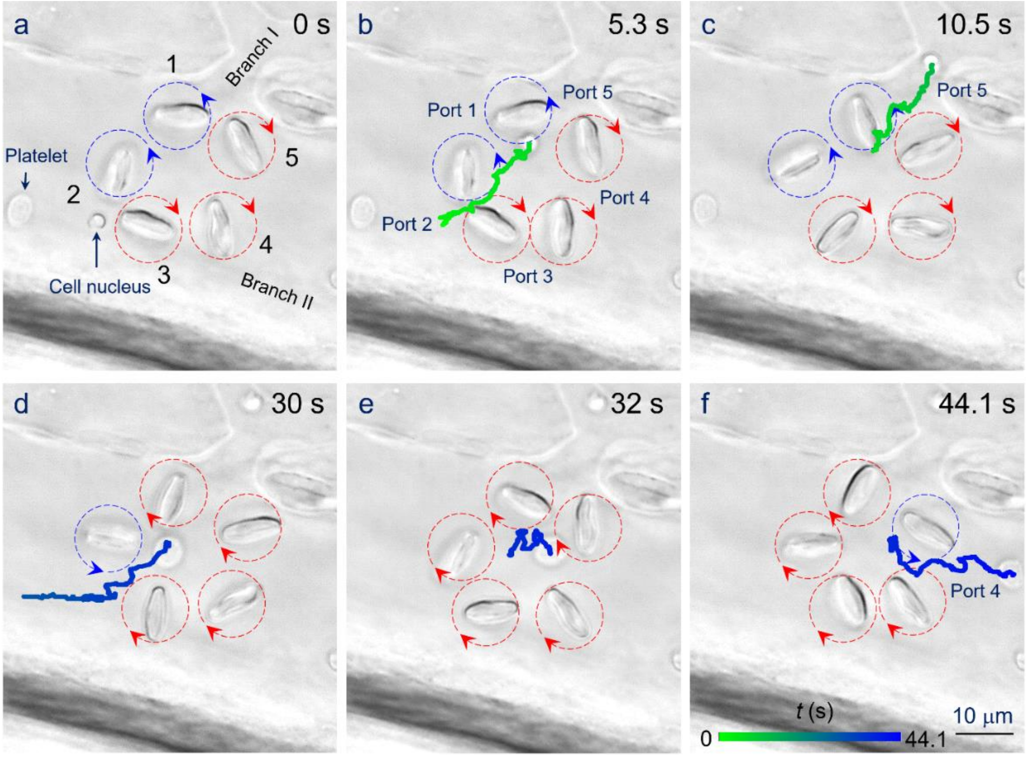

Figure S12. Optical microscopic images for routing the cell nucleus and platelet respectively towards the port 5 and 4, with the detiled motion trajectory indicated by the time-mapping colorful curve. (**a**) At t = 0 s, one cell nucleus and one platelet were located at the port 2 of the RBC microrouter. (**b**) By rotating RBC 1&2 anticlockwise while RBC 3, 4&5 clockwise, the cell nucleus was imported into the microrouter from port 2 at t = 5.3 s. (**c**) The cell nucleus was then exported from port 5 and then entered into the branch I at t = 10.5 s. (**d**) By rotating RBC 2 anticlockwise while RBC 1, 3, 4&5 clockwise, the platelet was imported into the RBC microrotor at t = 30 s. (**e**) The RBC microrotors were then regulated to rotate in the anticlockwise direction, under which the platelet was tranported towards the port 4. (**f**) By rotating RBC 4 anticlockwise while RBC 1, 2, 3&5 clockwise, the platelet was exported from the microuter successfully and then entered into the branch II.

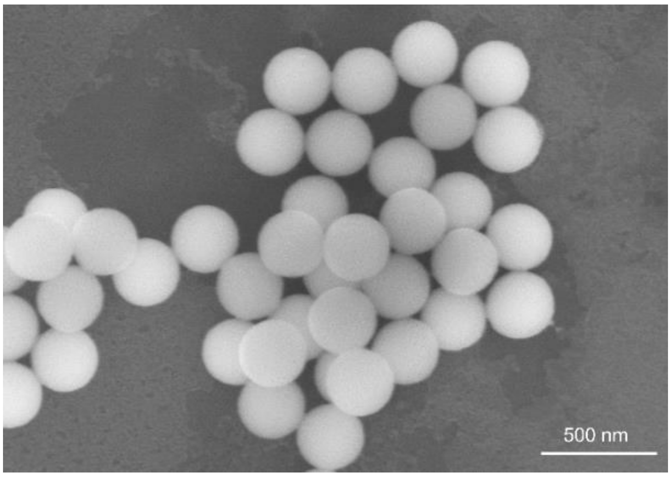

Figure S13. Scanning electron microscope image of the MSNs.

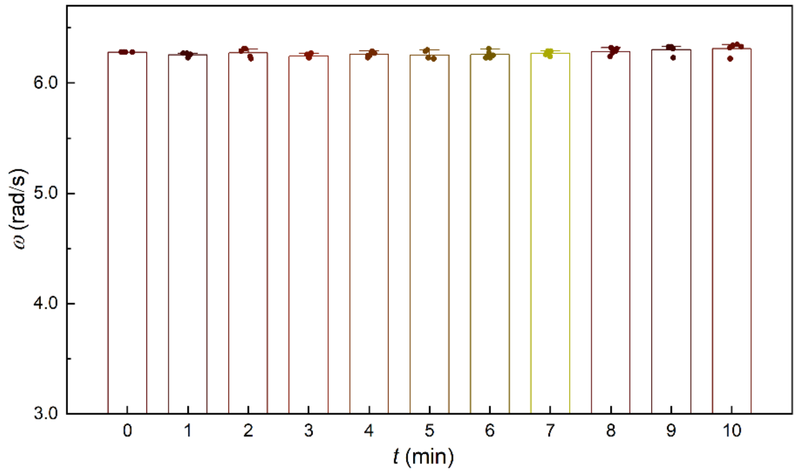

Figure S14. The quantitative characterization for the rotation stability of RBC microrouter. To further demonstrate the stability of the RBC rotation under optical manipulation, the rotation velocity for the RBC microrotor was tested for a continuous rotation of 10 minutes (n = 5). The results indicated that the RBC microrotor exhibited a high stability during the long-term rotation without a significant degradation, thus providing a great potential for its long-term in vivo applications.

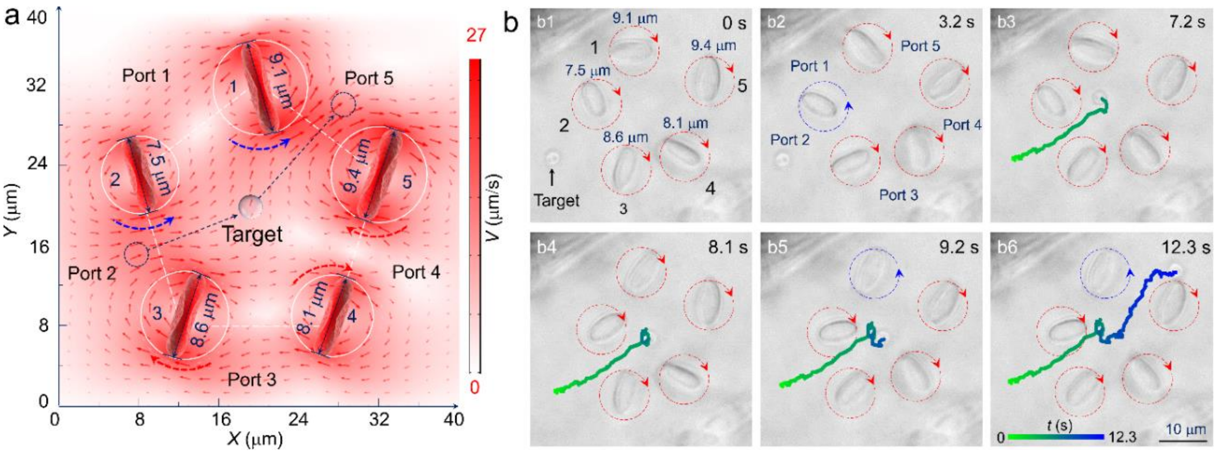

Figure S15. (a) Simulated microflow field generated by the RBC microrouter consisted of five different RBCs, with the diameters of 9.1, 7.5, 8.6, 8.1, and 9.4 μm, respectively. By rotating RBC 1&2 anticlockwise and 3, 4&5 clockwise, the obtained microflow field would still tend to import the target from port 2 and then export it from port 5. (**b**) Optical microscopic images for routing the target using the RBC microrouter consisted of five different RBC microrotors. (**b1**) At t = 0 s, one RBC microrouter was assembled while one target was located at the entrance of port 2. (**b2**) Afterwards, the RBC 2 was regulated to rotate in the anticlockwise direction. (**b3-b5**) Consequently, the target was imported into the microrouter via port 2 successfully, followed by the rotation around the center along the anticlockwise direcrion. (**b6**) Once the target approached port 5, the rotation direction of RBC 1 was switched and the target was exported from the microrouter through port 5.

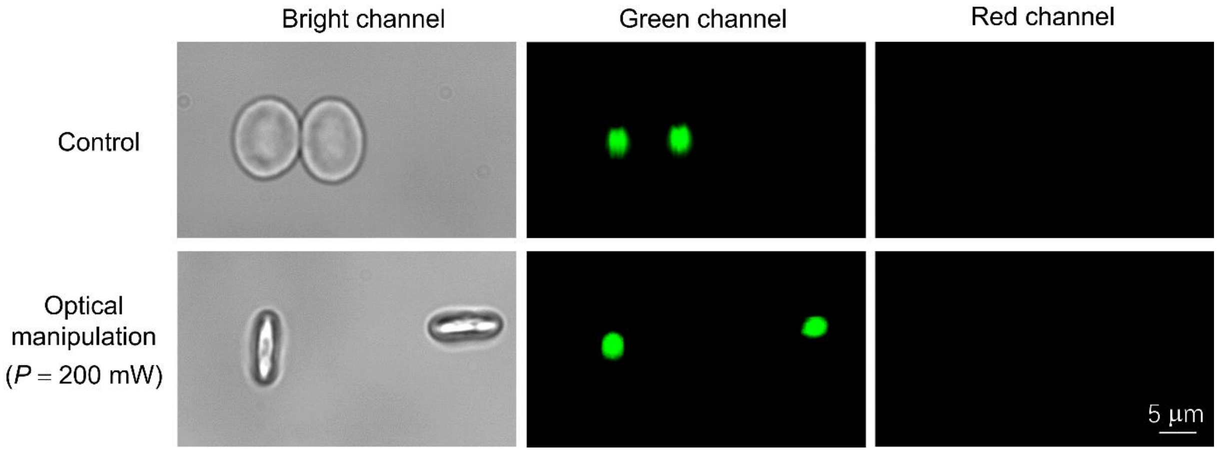

Figure S16. Acridine orange/ethidium bromide (AO/EB) staining for characterizing the potential cell damage of the optically manipulated RBCs. The alive cells only exhibit green fluorescence while red fluorescence signals will also be detected for the apoptotic cells. Notably, two RBCs trapped and rotated by the laser beam (P = 200 mW) exhibited only green fluorescence staining, which was similar with the control group without optical manipulation, indicating no obvious cell damage under the current experimental condition.

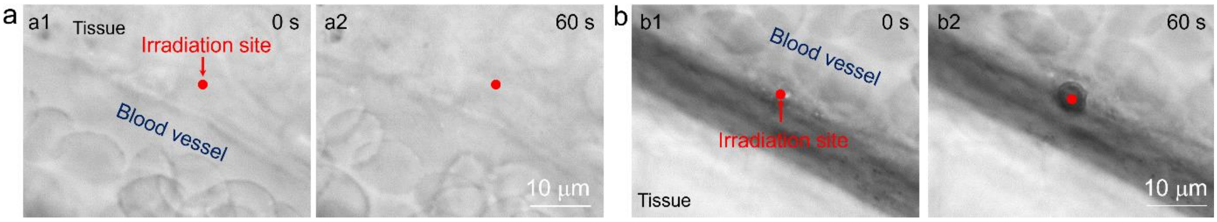

Figure S17. (**a**) Optical microscopic images of the ordinary tissue before and after laser irradiation for 60 s. (**b**) The melanin-accumulated region suffered from a significant burning after the laser irradiation of 60 s.
